# Ribavirin Enhances the Action of Interferon-α against Hepatitis C Virus by Promoting the p53 Activity through the ERK1/2 Pathway

**DOI:** 10.1371/journal.pone.0043824

**Published:** 2012-09-04

**Authors:** Wei-Liang Liu, Hung-Chih Yang, Wen-Cheng Su, Chih-Chiang Wang, Hui-Ling Chen, Hurng-Yi Wang, Wen-Hung Huang, Ding-Shinn Chen, Ming-Yang Lai

**Affiliations:** 1 Graduate Institute of Clinical Medicine, and Department of Internal Medicine, National Taiwan University College of Medicine and Hospital, Taipei, Taiwan; 2 Department of Microbiology, National Taiwan University College of Medicine, Taipei, Taiwan; 3 Hepatitis Research Center, National Taiwan University Hospital, Taipei, Taiwan; University of Tennessee Health Science Center, United States of America

## Abstract

**Background/Aims:**

Ribavirin significantly enhances the antiviral response of interferon-α (IFN-α) against Hepatitis C virus (HCV), but the underlying mechanisms remain poorly understood. Recently, p53 has been identified as an important factor involving the suppression of HCV replication in hepatocytes. We, therefore, decided to investigate whether and how ribavirin inhibits the replication of HCV by promoting the activity of p53.

**Methods:**

HepG2 and HCV replicons (JFH1/HepG2) were utilized to study the relationship between ribavirin and p53. The effect of ribavirin on cell cycles was analyzed by flow cytometry. The activation of p53 and the signaling pathways were determined using immunoblotting. By knocking down ERK1/ERK2 and p53 utilizing RNA interference strategy, we further assessed the role of ERK1/2 and p53 in the suppression of HCV replication by ribavirin in a HCV replicon system.

**Results:**

Using HepG2 and HCV replicons, we demonstrated that ribavirin caused the cell cycle arrest at G1 phase and stabilized and activated p53, which was associated with the antiviral activity of ribavirin. Compared to either ribavirin or IFN-α alone, ribavirin plus IFN-α resulted in greater p53 activation and HCV suppression. We further identified ERK1/2 that linked ribavirin signals to p53 activation. More importantly, knockdown of ERK1/2 and p53 partially mitigated the inhibitory effects of ribavirin on the HCV replication, indicating that ERK1/2-p53 pathway was involved in the anti-HCV effects of ribavirin.

**Conclusion:**

Ribavirin stimulates ERK1/2 and subsequently promotes p53 activity which at least partly contributes to the enhanced antiviral response of IFN-α plus ribavirin against HCV.

## Introduction

Ribavirin exhibits a broad-spectrum antiviral activity against DNA and RNA viruses [1]. Ribavirin can significantly enhance the effects of IFN-α on suppression of Hepatitis C virus (HCV) replication although ribavirin alone only induces a moderate and transient decline of viral loads and normalization of serum aminotransferase in a portion of HCV-infected patients [2], [3]. Several mechanisms have been proposed to explain the antiviral activities of ribavirin. First, ribavirin monophosphate has been shown to act as a competitive inhibitor of inosine-5′-monophosphate dehydrogenase (IMPDH), a cellular enzyme required for the de novo synthesis of guanosine triphosphate. Depletion of guanosine triphosphate may inhibit viral replication indirectly [4]–[6]. Second, ribavirin may have an immunomodulatory effect by favoring the T-helper 1 cytokine response [7]–[9]. Third, ribavirin triphosphate may directly inhibit the HCV-RNA–dependent RNA polymerase by acting as a substrate and cause mis-incorporation or premature primer chain termination, leading to inhibition of viral replication [10], [11]. Forth, ribavirin can act as a mutagen, causing lethal mutagenesis and error catastrophe [12]–[15]. Furthermore, ribavirin has been shown to enhance the expression of interferon-stimulated genes [16], [17], partly contributing to the enhanced antiviral response in combination therapy with IFN-α and ribavirin. However, the detailed mechanisms concerning how ribavirin promotes the IFN signaling remains to be clarified.

p53, a tumor suppressor gene, is the most frequent target of genetic alternations in human cancers. Activation of p53 leads to cell cycle arrest, apoptosis, DNA repair and senescence [18], [19]. p53 can serve as a transcription factor and regulate many downstream genes. One of these genes, p21, regulates the cyclin-Cdk complexes to invoke G_1_ and G_2_-M growth arrest [20]. Another important target gene of p53 is Mdm2, which targets p53 for degradation via the ubiquitination pathway, promotes its nuclear export, and thus allows cell cycle progression [21]. Post-translational modifications of p53 by phosphorylation, acetylation, and sumoylation have been proposed to be important mechanisms in regulating the stability and functions of p53 [22]. Phosphorylation of serine 15 residue in the transactivation domain of p53 has been implicated in disruption of p53-Mdm2 interaction, leading to a decrease in p53 degradation and its subsequent stabilization and to an increase in p53-dependent transactivation activity [23].

Multiple serine/threonine kinases, including ATM, ATR, DNA-PK, have been implicated in the upstream signaling that results in p53 phosphorylation at serine 15 in vitro [24]. Recently, several reports have shown that the phosphorylation of p53 is mitogen-activated protein (MAP) kinases-dependent. The MAP kinase pathways are parallel cascades of structurally related serine/threonine kinases that serve to integrate numerous extracellular signals in regulation of cell proliferation, differentiation, stress response, and cell survival [25].

Ribavirin can restrict the biosynthesis of guanylates and inhibition of cell proliferation and differentiation through p53 [26]. Besides, as mentioned above, p53 plays an important role in the cell defense against virus infection [27]–[30]. Therefore, we speculate that ribavirin may stimulate the antiviral effect of p53 that contributes to the enhanced anti-HCV activity of the combination therapy with IFN-α and ribavirin. In this study, we provided the evidence that support this hypothesis and explored the mechanisms in regulating the p53 activity induced by ribavirin.

## Results

### Ribavirin induced cell cycle arrest at G1 phase

**Figure 1:**
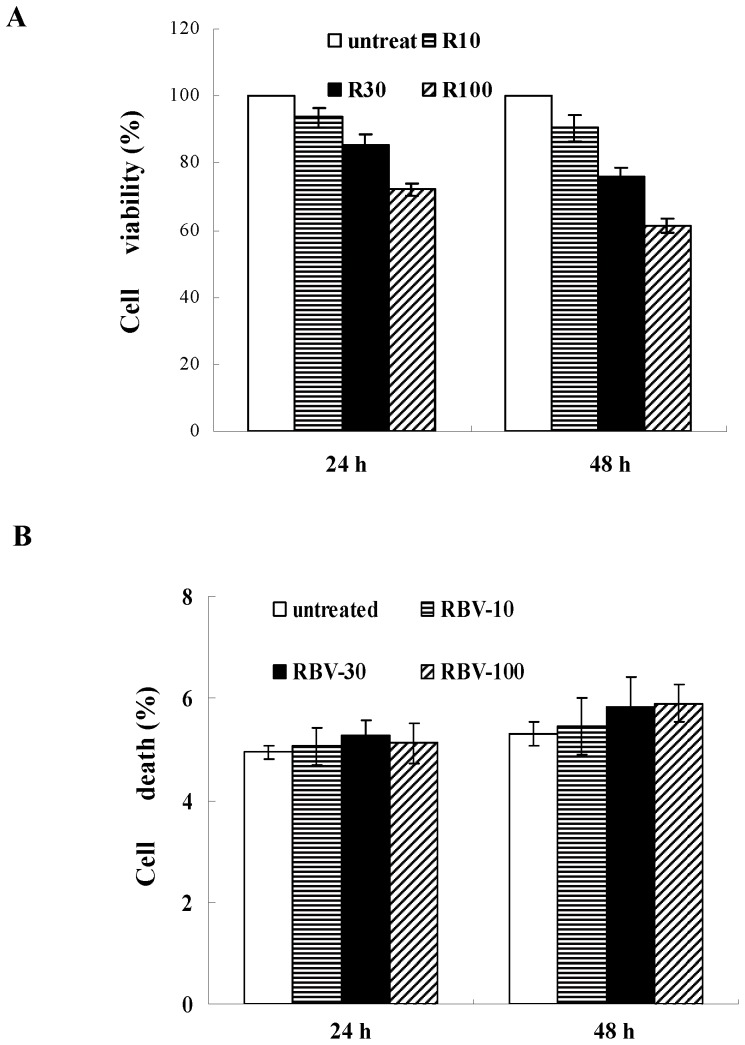
Ribavirin inhibits cell proliferation in HepG2 cells. (**A**) Cells were treated with the indicated amounts of ribavirin for 24 h and 48 h. The cells viability was determined by the MTT assay. (**B**) Cells were exposed to ribavirin for different time points and incubated with 5 µl FITC-conjugated Annexin V for 15 min in the dark at room temperature. Externalization of phosphatidylserine was identified by flow cytometry (FACScan). Data shown in (A) and (B) represent the mean ± s.e.m of five independent experiments. RBV-10: 10 µg/ml; RBV-30: 30 µg/ml; RBV-100: 100 µg/ml.

We first measured the cytotoxic effects of ribavirin on HepG2 cells by the MTT assay and annexin-V/propidium iodide labeling. Using the MTT assay, we found that ribavirin treatment reduced the number of viable cells in a dose-dependent manner, indicating that ribavirin either suppressed cell proliferation or induced cell death (Fig. 1A). However, the annexin-V assay demonstrated that ribavirin did not significantly increase cell death (Fig. 1B). Taken together, these results suggest that the decline of cell viability resulted from the inhibition of cell proliferation, instead of induction of cell death.

**Figure 2:**
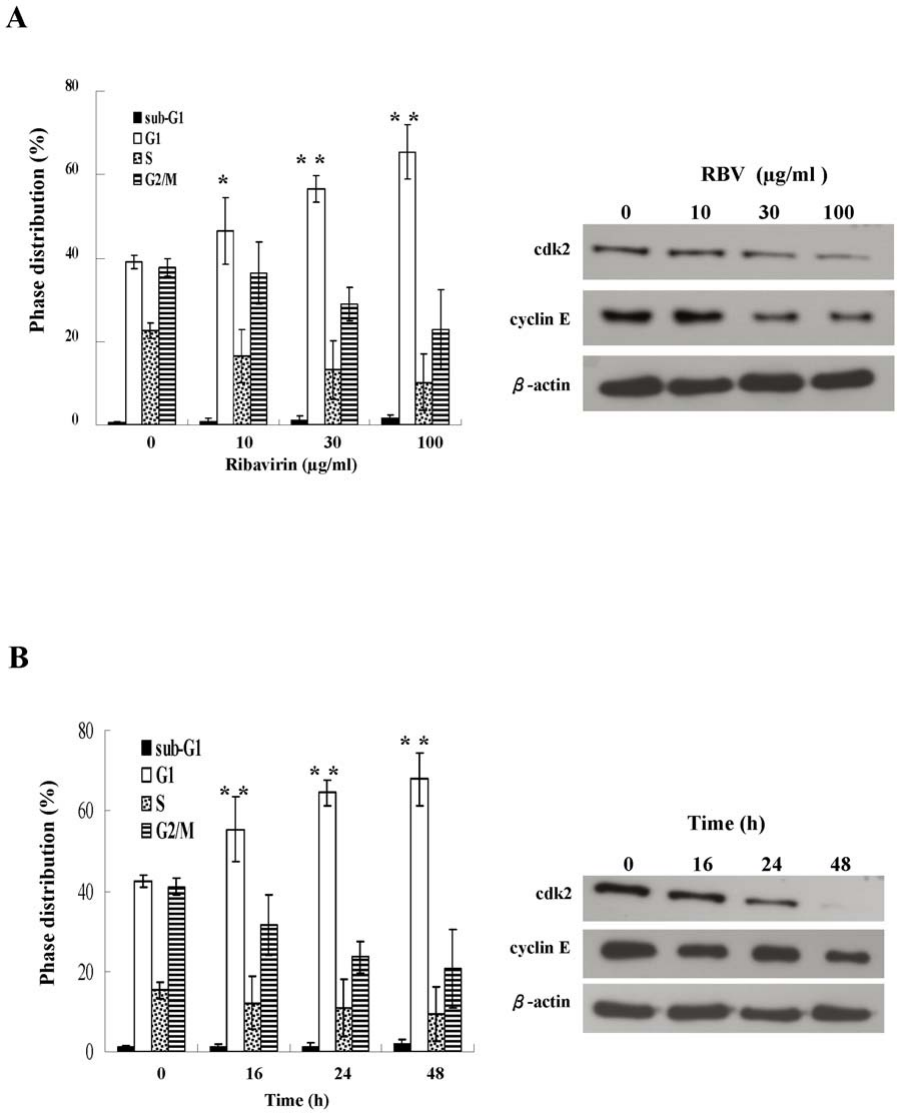
The profiles of cell cycle phases in ribavirin-treated cells. HepG2 cells were treated with the (**A**) indicated doses of ribavirin (0∼100 µg/ml) for 24 h, or (**B**) with the 100 µg/ml ribavirin for indicated periods of time (0∼48 h). Cells were stained with propidium iodide and then subject to flow cytometric analysis to determine the effect of ribavirin on cell cycle distribution. The percentage of cells in G1, S, and G2/M phase of cell cycle and the percentage of cells in sub-G1 (apoptosis) peak were calculated using CellQuest software. Cells were also analyzed by immunoblotting for the cell cycle regulators: cdk2 and cyclin E. Indicated values are means ± s.e.m of three independent experiments, in each of which triplicate samples were measured. The G1 population of each group was compared to those of the reference groups which are untreated cells in (A) and 0 hour in (B). ^*^, *p* = 0.04, ^**^, *p*<0.01. RBV: ribavirin.

Next, we investigated whether the inhibition of cell growth by ribavirin was caused by the cell cycle arrest. We observed that the cells receiving 10, 30 and 100 µg/ml of ribavirin exhibited 46.4%, 56.6% and 65.4% cell cycle arrest at G1 phase, respectively. Compared to untreated cells which exhibited 39.1% cell cycle arrest, a significantly higher portion of ribavirin-treated cells arrested at the G1 phase (Fig. 2A). In the time-course studies, ribavirin induced the cell cycle arrest at the G1 phase within 16∼48 h (Fig. 2B). We also examined the effect of ribavirin on the cell cycle regulatory molecules that affect the G1 phase. We demonstrated that ribavirin decreased the expression of cyclin E and CDK2, which was correlated with the cell cycle arrest (Fig. 2A and 2B). Moreover, we also showed that ribavirin inhibited the kinase activities of CDK2, indicated by the reduced interaction between CDK2 and its substrate histone H1 following ribavirin treatment (data not shown). Taken together, these results suggest that ribavirin induces the cell cycle arrest in a dose- and time-dependent manner.

### Ribavirin enhanced p53 protein stability and activity

**Figure 3:**
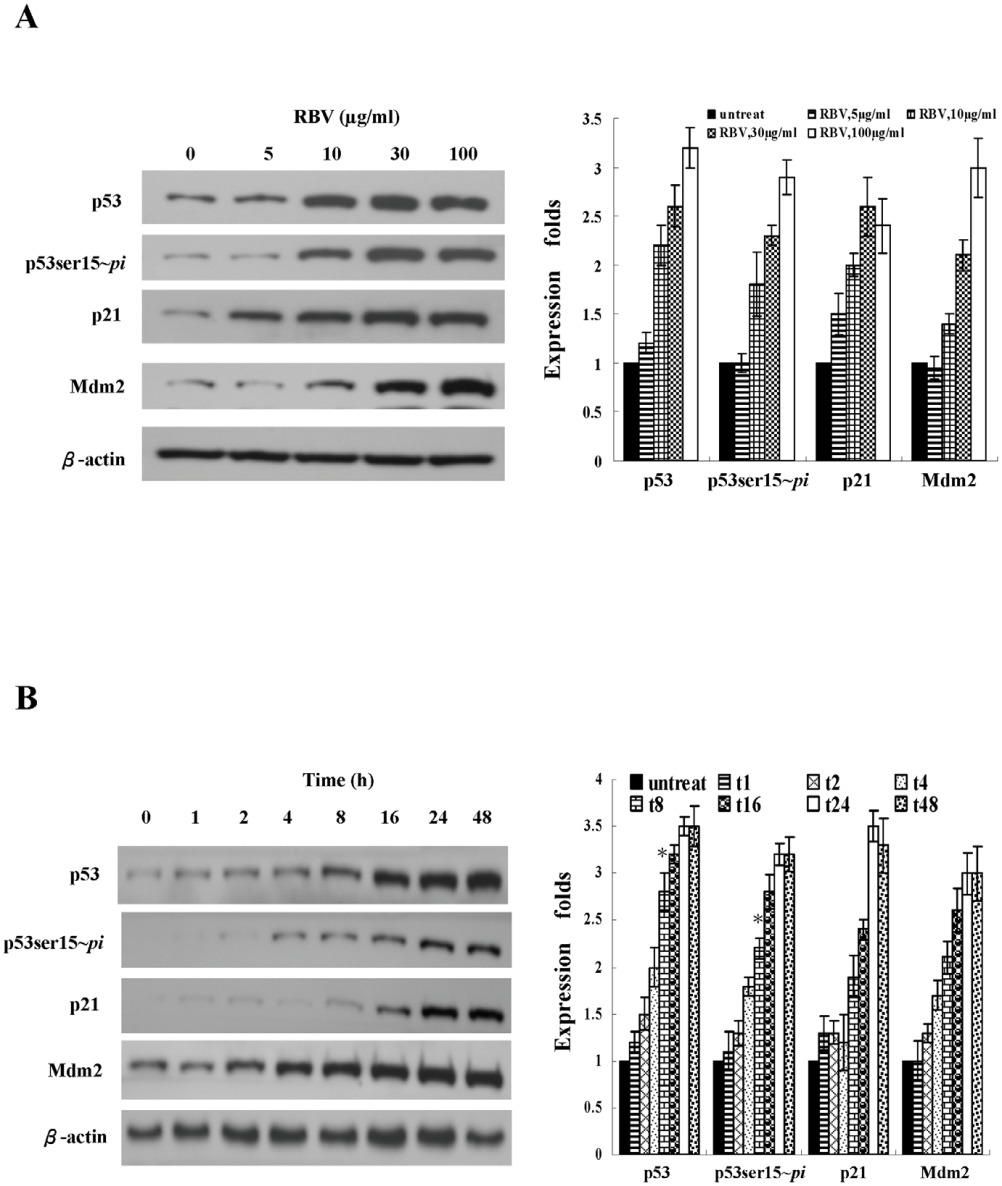
Ribavirin induces the p53 protein expression and phosphorylation. (**A**) Dose-dependent expression and phosphorylation of p53 induced by ribavirin. HepG2 cells were treated with the indicated doses of ribavirin (0, 5, 10, 30, 100 µg/ml) for 24 h. (**B**) The time-course p53 expression during ribavirin treatment. HepG2 cells were treated with 30 µg/ml ribavirin and harvested at the indicated time points (0∼48 h). The cell extracts were subject to immunoblot analysis for phosphorylated p53 at Ser15 (p53ser15∼*pi*), p53, p21, Mdm2. The Western blot experiments were repeated for four times and the representative results are shown here. The band intensity of each protein in each experiment was determined by densitometry. The expression fold change of proteins were calculated by dividing the band intensity of the indicated proteins, normalized to that of the loading control β-actin, with that observed in the samples receiving no ribavirin treatment or at zero time of ribavirin treatment. The results are illustrated by the bar graph located on the right side of the Western blot analysis. Indicated values are means ± s.e.m of four independent experiments. RBV: ribavirin.

**Figure 4:**
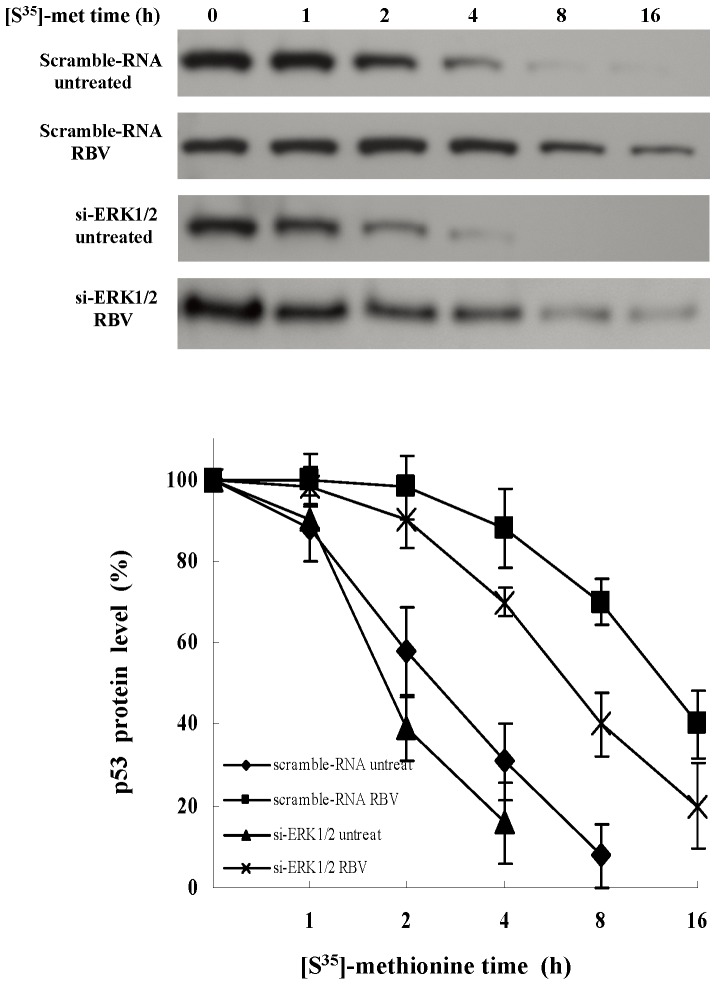
The half-life of p53. The scrambled- and ERK1/2- siRNA transfected HepG2 cells were growing in the DMEM devoid of L-methionine for 3 h and then incubated with 200 µCi/ml of [S^35^]-methionine for 4 h. After removal of the medium, cells were treated with or without ribavirin (100 µg/ml) for the indicated times. At the end of the treatment period, cells were harvested and lysed. Total cell extracts were immunoprecipitated with anti-p53 antibody and subjected to SDS-PAGE for fluorography. The level of [S^35^]-labeled p53 was quantified. The data represented 4 independent experiments which gave similar results. RBV: ribavirin.

p53 is involved in regulating cell cycle arrest, so it is possible that p53 may play a role downstream to ribavirin. Therefore, we further examined whether ribavirin could regulate the expression and activity of p53. Based on the literature and our previous study, we knew that the expression and function of p53 were impaired in several cell lines, like Huh7 (p53-mutant) and Hep3B (p53- null) [30]. In this study, we thus chose HepG2 cells, which express wild-type p53. Following different doses of ribavirin treatment, we observed the upregulation of both the total and phosphorylated p53 proteins in HepG2 cells (Fig. 3A). The time-dependent effect of ribavirin on the induction of p53 protein was examined at a fixed concentration of 30 µg/ml. A significant increase in the total and phosphorylated p53 proteins was first detectable at 8 h after the addition of ribavirin (Fig. 3B). Previously, we demonstrated that ribavirin did not stimulate p53 transcription [30]. Therefore, it was likely that the increased expression of p53 protein was due to either its increased protein translation or reduced degradation. We also used the pulse-chase approach to assess the p53 degradation. The half-life (t_1/2_) of the p53 protein in the untreated cells was 2.6 hrs, whereas the t_1/2_ of p53 in ribavirin-treated cells was prolonged to 12.2 hrs (Fig. 4). In addition, the p53-dependent proteins, p21 and Mdm2, were also increased (Fig. 3A and 3B). This phenomenon was not specific for HepG2 cells because we also observed the same results in primary human hepatocytes (data not shown). These results reveal that ribavirin stabilizes and activates p53 protein in a dose- and time-dependent manner.

### Ribavirin enhanced the p53-dependent transcription

**Figure 5:**
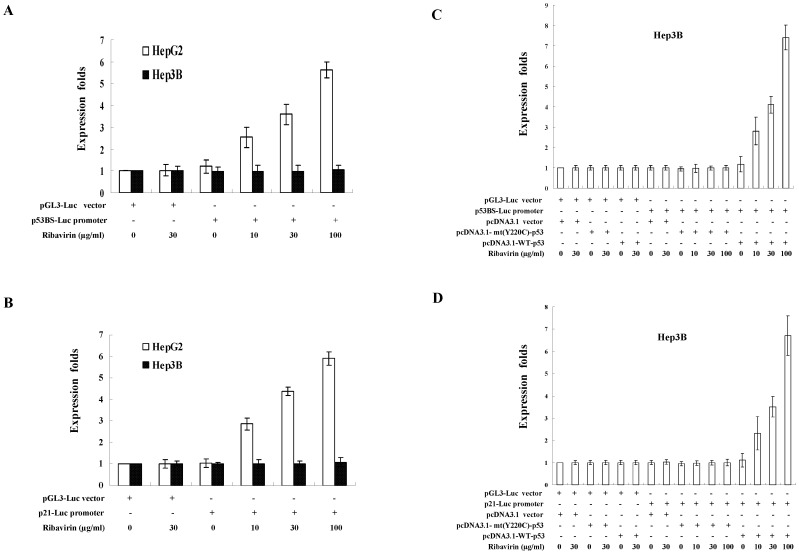
The p53-dependent transcriptional activity enhanced by ribavirin. HepG2 and Hep3B (p53-deficient) cells were transfected with (**A**) p53BS-Luc reporter or (**B**) p21-Luc reporter After transfection, cells were treated with the indicated concentration of ribavirin for 24 h. The pRL-TK plasmid was co-transfected for the purpose of normalization. (C) p53BS-Luc reporter or (D) p21-Luc reporter was co-transfected with either wild-type p53-expression vector, mutant p53 (Y220C) or the control vector pcDNA3.1 into Hep3B cells. Cells were then treated with indicated concentrations of ribavirin for 24 hours. The expression folds in (**A**)∼(**D**) were shown compared to that observed with the control reporter pGL3-Luc vector, after normalization with expression levels of the internal control pRL-TK. Each result represent the mean ± s.e.m of three independent experiments, in each of which triplicate samples were measured.

In order to confirm that the transcriptional activity of p53 was also enhanced by ribavirin, we performed p53-dependent reporter assays in HepG2 cells transfected with two different p53-dependent luciferase-reporter plasmids, the p53BS-Luc reporter and the p21-Luc reporter. Following the treatment of 10∼100 µg/ml ribavirin, the luciferase activity of the p53BS-Luc reporter was increased by 2.5- to 5.6-fold (Fig. 5A). The p21-Luc reporter was activated by 2.8- to 5.9-fold with the treatment of 10∼100 µg/ml ribavirin (Fig. 5B). In contrast, we did not observe the induction of p53-dependent transcription by ribavirin in the Hep3B cells, which expressed no p53. However, the inducing ability of ribavirin for these two reporter constructs could be restored in Hep3B cells when they were transfected with the wild-type p53-expressing vector, but not with the Y220C mutant of p53-expressing vector (Fig. 5C and 5D). In addition, we also confirmed that ribavirin increased the mRNA levels of endogenous p53-dependent genes Mdm2 and p21. However, knockdown of p53 abolished the upregulation of Mdm2 and p21 transcription by ribavirin (Fig. S1). Therefore, we concluded that the transcriptional activity of p53 was enhanced by ribavirin.

### Inhibition of viral gene expression and replication of HCV replicons by ribavirin were associated with p53 activation

**Figure 6:**
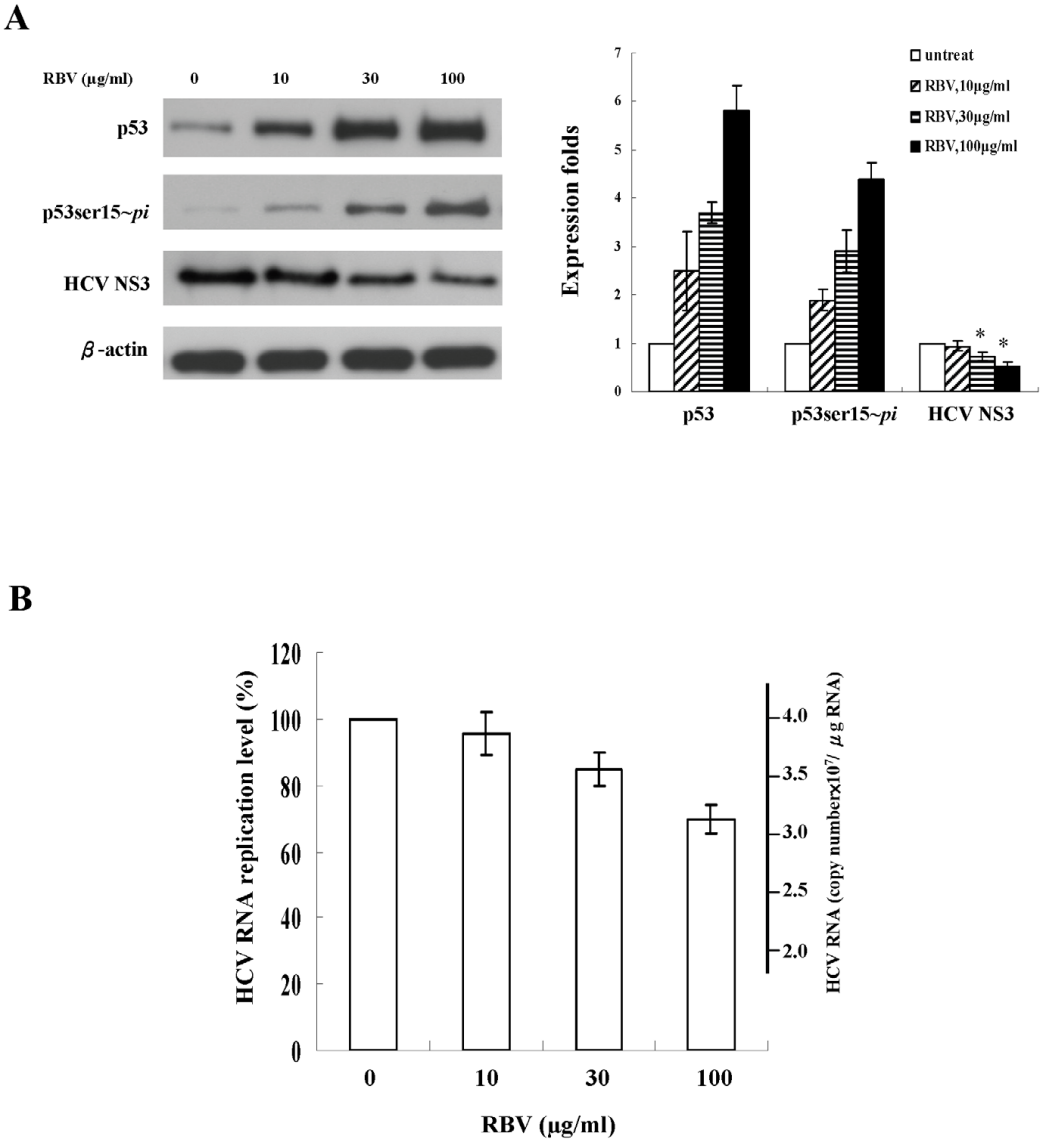
The effect of ribavirin on HCV replication in HCV replicons. (**A**) Replicons were treated with the indicated concentration of ribavirin for 48 h. The expression of total p53, phosphorylated p53 at Ser15 (p53ser15∼*pi*) and HCV NS3 viral proteins was analyzed by immunoblotting. The experiment was independently repeated 5 times and one representative result was shown here. The band intensity of each protein in each experiment was determined by densitometry. The expression fold change of proteins were calculated by dividing the band intensity of the indicated proteins, normalized to that of the loading control β-actin, with that observed in the samples receiving no ribavirin treatment. The results are illustrated by the bar graph located on the right side of the Western blot analysis. Each result represents the mean ± s.e.m of 5 independent measurements and was considered statistically significant at *P*<0.05. The HCV NS3 protein of each group was compared to those of the reference groups which are untreated cells. ^*^, *p*<0.05. (**B**) HCV RNA levels were determined by quantitative RT-PCR. Each result represents the mean ± s.e.m of 5 independent measurements and was considered statistically significant at *P*<0.05 versus the untreated group. RBV: ribavirin.

Previous studies have shown that p53 plays a role in the suppression of viral replication [27]–[30]. To examine whether p53 activity is associated with the suppression of HCV replication by ribavirin, we analyzed the expression of HCV NS3 and p53 protein in the ribavirin-treated replicon cells (JFH1/HepG2) using immunoblotting. Ribavirin reduced the levels of the HCV NS3 protein, which were inversely correlated with the levels of phosphorylated p53 (Fig. 6A). By quantitative RT-PCR, we further determined the intracellular HCV RNA levels of the replicons. Treatment with 30 and 100 µg/ml ribavirin resulted in 15% and 30% reduction of the replicating HCV RNA levels, respectively (Fig. 6B). These data indicate that inhibition of viral protein expression and replication of HCV replicons by ribavirin are associated with the upregulation of phosphorylated p53.

### Knockdown of p53 by shRNA significantly attenuated the inhibitory effects of ribavirin on HCV replication

**Figure 7:**
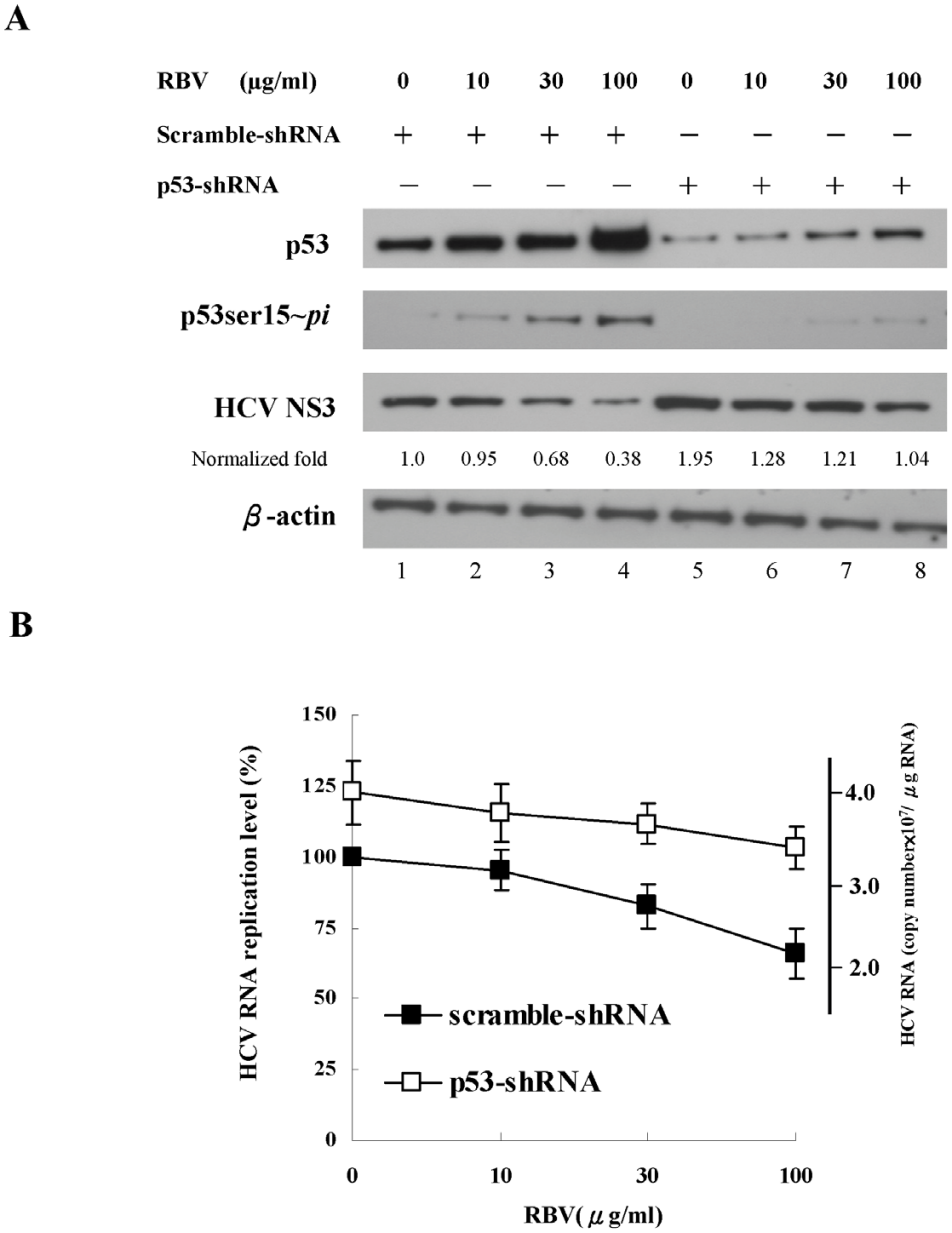
The impact of p53 silencing on HCV replication in replicon cells. Lentiviral vector-mediated silencing of endogenous p53 in replicon cells (JFH1/HepG2) treated with the indicated concentration of ribavirin for 48 h. (**A**) The expression of total p53, phosphorylated p53 at Ser15 (p53ser15∼*pi*) and HCV NS3 viral proteins was analyzed by immunoblotting. The Western blot was independently repeated five times and one representative result was shown here. The band intensity of each protein in each experiment was determined by densitometry. The expression level change of proteins were calculated by dividing the band intensity of the indicated proteins, normalized to that of the loading control β-actin, with that observed in the samples receiving scramble-shRNA treatment. (**B**) HCV RNA levels were measured by quantitative RT-PCR. Each result represents the mean ± s.e.m. of 5 independent determinations and is considered significant at *P*<0.05. RBV: ribavirin.

To further prove the critical role of p53 activity in inhibiting HCV replication in the replicon system, we used p53-shRNA to silence the p53 expression. In Figure 7, p53-shRNA dramatically reduced the expression of the total and phosphorylated p53 proteins in replicon cells. Moreover, knockdown of p53 by shRNA resulted in 95% increase in the HCV NS3 viral protein compared to the scrambled-shRNA treatment (Fig. 7A, lane 5 vs. lane 1). Following the treatment with 100 µg/ml ribavirin, the HCV NS3 protein increased about 180% in the p53-shRNA transfected cells compared to those transfected with scrambled-shRNA (Fig. 7A, lane 8 vs. lane 4).

We also examined whether subgenomic HCV RNA levels were influenced by knockdown of p53. We used the quantitative RT-PCR to measure the levels of HCV RNA in the p53-shRNA treated cells receiving ribavirin treatment. In the replicon cells transfected with scrambled-shRNA, ribavirin treatment (100 µg/ml) could reduce the HCV RNA replication by 35%, whereas we observed only 20% reduction of HCV RNA levels in p53-shRNA transfected cells receiving ribavirin treatment (Fig. 7B). To quantitatively demonstrate the critical role of p53 in the antiviral activity of ribavirin, we compared the dose-dependent suppressive activity of ribavirin in both scrambled- and p53- shRNA treated cells using an ANOVA test. P53 and ribavirin were treated as different factors and both of them were highly significant (p<10^−3^). In addition, the term of interaction between ribavirin and absence of p53 is also significant (p = 0.04), indicating that that the antiviral effect of ribavirin was stronger in the presence of p53 than that in the absence of p53 (Table S1). These findings demonstrated that abolishment of the endogenous p53 activity by p53-shRNA could at least partly, although not completely, restore HCV replication in ribavirin-treated HCV replicon cells. All of these results indicate that p53 indeed exhibits inhibitory effects on HCV replication, and further confirm the role of p53 in the antiviral activity of ribavirin.

### p53 activity contributed to the greater activity against HCV replication by combination therapy with ribavirin and IFN-α

**Figure 8:**
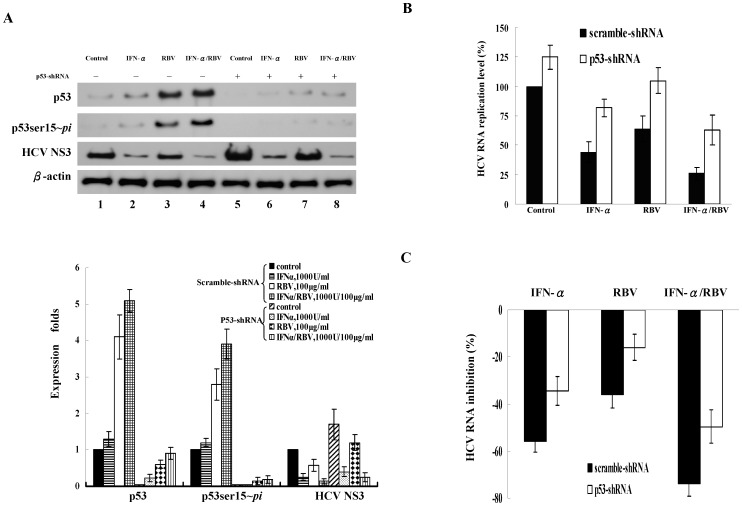
The effect of ribavirin (RBV) combined with IFN-α on p53 activity and the suppression of HCV replication. The scrambled-shRNA and p53-shRNA transduced replicon cells (JFH1/HepG2) were treated with IFN-α (1000 U/ml), RBV (100 µg/ml) or the combined treatment with IFN-α and RBV for 48 h. (**A**) The expression of total p53, phosphorylated p53 at Ser15 (p53ser15∼*pi*) and HCV NS3 viral protein was analyzed by immunoblotting. The Western blot was independently repeated five times and one representative result was shown here. Levels of p53, phosphorylated p53 and HCV NS3 were quantified by densitometry analysis. The expression fold change of proteins were calculated by dividing the band intensity of the indicated proteins, normalized to that of the loading control β-actin, by that observed in the samples receiving scramble-shRNA treatment. (**B**) HCV RNA replication levels were determined by quantitative RT-PCR. (**C**) The percentage of HCV RNA inhibition was calculated as the HCV RNA levels of individual samples (IFN, RBV or IFN/RBV-treated samples) subtracted those of control samples (untreated samples) and then was divided by those of control samples using the data from (B). Each result represents the mean ± s.e.m. of 5 independent determinations and is considered significant at *P*<0.05. Control: untreated, IFN: INF-α, RBV: ribavirin.

To investigate whether p53 also plays a role in the synergistically antiviral activity of combination therapy with ribavirin plus IFN-α against HCV, we silenced the expression of p53 in HCV replicon cells and subsequently treated them with IFN-α, ribavirin, or IFN-α plus ribavirin. In the replicon cells transduced with the scrambled-shRNA, we found that IFN-α, ribavirin and IFN-α plus ribavirin enhanced the levels of phosphorylated p53 and decreased the expression of the HCV NS3 protein (Fig. 8A, lane 1∼4). In the replicon cells transduced with p53-shRNA, the phosphorylated p53 was barely detectable and the HCV NS3 viral protein (Fig. 8A, lane 5∼8) and HCV RNA levels were increased compared to cells receiving scrambled-shRNA (Fig. 8B). Ribavirin combined with IFN-α has a greater effect on the upregulation of phosphorylated p53 that exhibited greater suppression of HCV RNA replication. In addition, we found that the suppression of HCV RNA levels by ribavirin in the presence of scrambled- or p53-shRNA was 36% and 16% (p<0.05), respectively (Fig. 8C). This indicated that knockdown of p53 did affect the antiviral effects of ribavirin. Consistently, the suppression of HCV RNA levels by IFN-α plus ribavirin in the presence of scrambled or p53-shRNA was 74% and 50% (p<0.04), respectively. This further supported the antiviral role of ribavirin.

### Induction of ERK1/2 phosphorylation by ribavirin correlated with p53 phosphorylation and suppression of HCV replication

**Figure 9:**
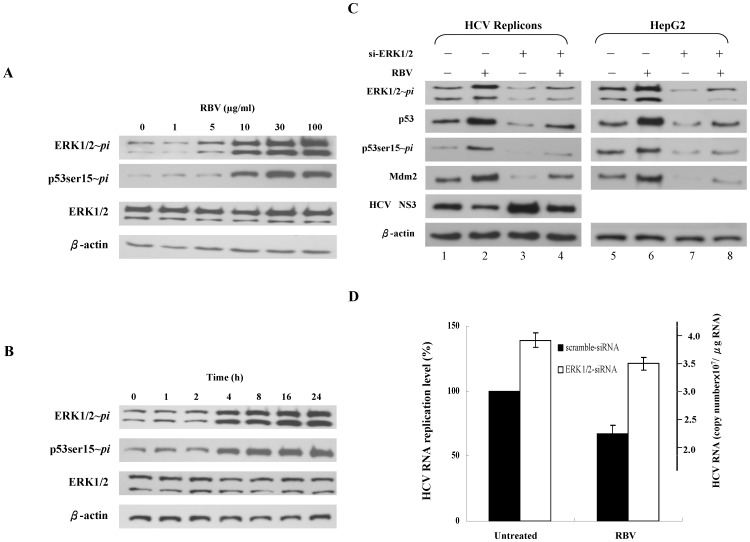
Induction of ERK1/2 phosphorylation by ribavirin correlated with p53 phosphorylation and suppression of HCV replication. (**A**) Dose-dependent phosphorylation of ERK1/2 by ribavirin. HepG2 cells were treated for 24 h with the indicated doses of ribavirin. (**B**) The association of ribavirin-induced ERK1/2 phosphorylation with p53 phosphorylation in a time-course analysis. HepG2 cells were untreated or treated with 30 µg/ml ribavirin and then harvested at the indicated time points. In addition, we used small-interference RNA (siRNA) to knockdown ERK1/ERK2 to assess the role of ERK1/2 and p53 in the suppression of HCV replication. (**C**) Cells were transduced with ERK1/2-siRNA (100 nM) and scrambled-siRNA for 48 h and treated with ribavirin (100 µg/ml) for 24 h in HCV replicon cells (JFH1/HepG2) and HepG2 cells. Cells were lysed, and analyzed for the expression of phosphorylated ERK1/2 (ERK1/2∼*pi*), phosphorylated p53 at Ser15 (p53ser15∼*pi*), p53, ERK1/2, Mdm2 and HCV NS3 proteins by immunoblotting. (**D**) HCV RNA expression levels of scramble-siRNA- or si-ERK1/2-transduced cells with/without ribavirin treatment were determined using quantitative RT-PCR. Each result represents the mean ± s.e.m of 5 independent measurements and is considered statistically significant at *P*<0.05. Control: no ribavirin treatment, RBV: ribavirin.

p53 activity can be regulated by a number of signaling pathways, among which MAP kinases play a role in stimulating the phosphorylation of p53 [25]. We, therefore, explored whether ribavirin could enhance the phosphorylation of MAP kinases, like ERK1/2, p38 and JNK. We found that phosphorylation of ERK1/2, as measured by immunoblotting, was enhanced by ribavirin in a dose-dependent manner in HepG2 cells, but the total protein levels of ERK1/2 showed no significant changes (Fig. 9A). In the kinetic studies, the ERK1/2 phosphorylation was readily detected at 4 h after ribavirin treatment (Fig. 9B), and peaked at 8 h to 24 h. However, we observed no significant changes of the total protein levels of ERK1/2 over the corresponding time course (Fig. 9B). Interestingly, the phosphorylation of ERK1/2 was correlated well with the phosphorylation of p53. On the contrary, the activity of p38 kinase and JNKs were not significantly increased following ribavirin treatment (data not shown).

To further confirm the critical role of ERK1/2 activation in p53 phosphorylation and HCV suppression by ribavirin, we performed an ERK1/2 knockdown experiment. The ERK1/2-siRNA efficiently suppressed both ERK1 and ERK2 expression and also reduced the levels of phosphorylated p53 and Mdm2 protein compared to the scrambled-siRNA (Fig. 9C). Moreover, we found that silencing of ERK1/2 decreased the stability of p53 (Fig. 4) and increased the expression of HCV NS3 viral protein (Fig. 9C, lane 3 vs. lane 1). In addition, by measuring the HCV RNA levels, we found that knockdown of ERK1/2 led to the decrease of viral suppression by ribavirin in replicon cells (Fig. 9D). We also used PD98059, a MEK1/2 inhibitor, to suppress the ERK1/2 phosphorylation. We observed that PD98059 blocked the ribavirin-induced p53 phosphorylation at serine 15 and increased the expression of HCV NS3 proteins in HCV replicon cells (Fig. S2). Taken together, these findings demonstrate that ERK1/2 activity contributes to the ribavirin-induced stability and activity of p53, leading to the suppression of HCV replication.

## Discussion

We have shown that ribavirin induced the upregulation and activation of p53 in HepG2 and HCV replicon cells (JFH1/HepG2), which correlated well with the antiviral activity of ribavirin. Combined treatment with ribavirin and IFN-α exhibited a greater stimulation of p53 activity and suppression of HCV replication than IFN-α alone. Moreover, knockdown of p53 mitigated the suppressive effect of ribavirin on HCV replication. Last, we discovered that activation of ERK1/2 by ribavirin was responsible for the ribavirin-induced p53 activation and antiviral activity. Taken together, these results suggest that ribavirin activates ERK1/2 and in turn stimulates p53 activity that is required for the anti-HCV activity of ribavirin in the combination therapy.

p53 has been recently reported to play a critical role in the cellular innate defense against viruses, including HCV [28],[29]. In this study (Figs. 6 and 7), we observed a 35% inhibition of HCV RNA in the ribavirin-treated (100 µg/ml) HCV replicon cells in the presence of functional p53, whereas ribavirin only suppressed HCV replication by 20% when p53 was silenced. Knockdown of p53 in HCV replicon cells caused a significant reduction, although not complete abolishment, of the antiviral activity of ribavirin. Around 43% ((35%–20%)/35%×100%) suppression of HCV replication by ribavirin could be attributable to the action of p53. Consistently, knockdown of ERK1/2 also led to a significant decrease, but not complete elimination, of the suppression of HCV replication by ribavirin in replicon cells. Therefore, our results indicate an important role of ERK1/2-p53 pathway in the antiviral response of ribavirin against HCV and thus supported the previous observation of other researchers regarding the antiviral activity of p53 [27]–[30].

Prior studies have revealed that the p53 inhibits viral replication by enhancing the IFN signaling. Zhang *et al.* demonstrated that ribavirin potentiates the expression of RSV-induced IFN-stimulated response genes (ISGs) and thus enhances the antiviral activity [31]. In addition, Dharel *et al.* reported that p53 is a key regulator in IFN-based therapy against HCV [28]. Moreover, Munoz-Fontela *et al.* have shown that functional p53 contributes to innate immunity by enhancing IFN-dependent antiviral activity via up-regulation of IFN regulatory factor 9 (IRF9) [29]. Recently, we have also reported that ribavirin-induced p53 activation enhances the transcription of IRF9 and elevates ISRE-dependent signaling [30]. Therefore, ribavirin-induced p53 activity can inhibit HCV replication probably through upregulation of ISGs expression. The crosstalk between the IFNs and ribavirin through p53 seems critical for its antiviral activity and deserves further studies to resolve the detailed mechanisms.

Of note, it has been shown that ribavirin efficiently inhibits HCV replication in Huh7 cells, which harbor mutant p53 (Y220C). We confirmed that mutant p53 of Huh7 cells did not exhibit antiviral activity against HCV (Fig. S3). However, this result was in contrast to the Dharel's report, which showed that p53 Y220C mutant could still suppress HCV replication. To further address this issue, we also performed the p53 transcriptional reporter assay in Hep3B cells and found that the Y220C mutant of p53 did not activate p53-dependent transcription (Fig. 5C and 5D). This result was distinct from that of Dharel's study. We currently do not know what really caused these differential results between ours and Dharel's study, but it seems unlikely that p53 Y220C mutant suppresses HCV replication through its transcriptional activity. Solving this issue may require the further study using the full-length JFH-1 infectious clone in Huh7 cells with restoration of wild-type p53 expression. Actually, our result implies that ribavirin inhibits HCV probably through p53-independent pathways in Huh7 cells. Consistent with this, failure to completely shut down the antiviral activity of ribavirin by silencing ERK1/2 and p53 also suggests the existence of ERK1/2-p53-independent pathway(s) that contribute to the ribavirin-induced antiviral effects. Alternatively, it can be explained by the residual p53 activity due to the incomplete p53-knockdown in the HCV replicon cells. However, the latter explanation was less likely because we could only barely detect the expression of residual p53 in the cells transduced with p53 shRNA. Consistent with this observation, we previously showed that ribavirin enhanced the IFN signaling, including IRF9, through both p53-dependent and p53-independent pathways [30]. Several previous studies have also demonstrated other pathways are involved in the suppression of HCV replication, including PKR and p56, that may serve as candidates of p53-independent pathways [32]–[34]. These p53-independent antiviral pathways deserve further investigation in the future.

MAP kinases are reported to involve the upregulation of the p53 expression [25]. Here, we found that activation of ERK1/2, but not p38 and JNK, by ribavirin is required for maximal accumulation and activation of p53. Knockdown of ERK1/2 significantly inhibited ribavirin-induced p53 activation and subsequent antiviral activity against HCV, suggesting that ribavirin suppressed HCV replication at least partly through activation of ERK1/2-p53 pathway. In addition, the MEK1/2 inhibitor PD98059 also suppressed p53 activation and HCV replication, indicating that MEK1/2 or upstream signaling molecule c-Raf may be also involved in ribavirin-induced p53 activation and HCV inhibition. We further demonstrated that ribavirin-induced ERK1/2 activity regulates both the stability and function of p53. Ribavirin stimulated the expression of the p53-dependent gene Mdm2, which can bind to p53 and inhibit the activation of p53 by targeting p53 protein for degradation through the ubiquitin-dependent proteolytic pathway [35]. Thus, Mdm2 and p53 are considered to form an autoregulatory feedback loop in which p53 limits its own activation through the induction of Mdm2 [36]. Here we showed that, in response to ribavirin, ERK1/2 is important for phosphorylation of p53 protein at serine 15, which has been known to decrease the binding of Mdm2 to p53, and thus increase p53 stability [24], [25],[37]. Consistently, knockdown of ERK1/2 decreased the half-life of p53, further supporting this conclusion.

Although we clearly showed that ribavirin activated ERK1/2-p53 pathways and thus suppressed HCV replication, how ribavirin regulates the activity of ERK1/2 remains elusive. In addition, silencing of ERK1/2 failed to completely eliminate the antiviral activity, suggesting that in addition to ERK1/2, ribavirin may stimulate unknown kinases or cellular factors which can lead to activation of ERK1/2-p53-independent pathways. Further studies are required to clarify the detailed mechanisms regarding how ribavirin regulates ERK-dependent and ERK-independent activity.

Additionally, we previously demonstrated that ribavirin promotes p53 activity by enhancing the activity of mTOR [30]. Since both mTOR and EKR1/2 are involved in p53 activation, it is reasonably assumed that mTOR signaling may cross talk with ERK1/2 signaling in some way. Previously, it has been shown that ERK1/2 could affect the expression of mTOR [38], indicating the potential interaction between ERK1/2 and mTOR. However, the detailed mechanisms involving the interaction remain unclear and will be further investigated in the future.

Although the development of direct antiviral agents (DAAs), like HCV protease and polymerase inhibitors have revolutionized the treatment of HCV infection [39]–[42], combination therapy with ribavirin and pegylated IFN-α may still remain essential components of the current standard regimen. However, it remains unclear how ribavirin enhances the antiviral effect of IFN-α. In this study, we provide evidence that the anti-HCV activity of ribavirin is at least partly attributable to upregulation of p53 activity and is responsible for the enhanced antiviral action of IFN-α by ribavirin. Importantly, we also identify ERK1/2 as an upstream factor that regulates p53 activity during ribavirin treatment. These findings not only provide deeper insight into the molecular mechanisms involved in the ribavirin-enhanced antiviral activity of IFN-α, but also suggest a potential therapeutic strategy that aims to upregulate the p53 activity in order to improve the outcomes of IFN-based anti-HCV therapy.

## Materials and Methods

### Cell lines and preparation of cellular extracts

HepG2 (wild-type p53) and Hep3B (p53-null) were maintained in Dulbecco's modified Eagle's medium containing 10% fetal bovine serum (FBS) and penicillin, streptomycin and glutamine. HCV replicon cells (JFH1/HepG2) were maintained in the MEM containing 10% FBS and penicillin, streptomycin, glutamine, and G418 [43]. Cells were treated for 24 h with different concentrations of ribavirin (Sigma-Aldrich) or with 30 µg/ml ribavirin for varying periods of time. To extract proteins, cells were washed with phosphate-buffered saline (PBS) and homogenized in lysis buffer (1% NP-40, 50 mM Tris, pH 7.5, 150 mM NaCl, 1 mM EDTA, 1 mM Na_3_VO_4_ and protease inhibitor cocktail (Roche)).

### Cell viability and cell death assays

Cell viability was determined using the MTT assay kit (Promega). Cell death was detected by annexin-V/propidium iodide labeling and samples were analyzed using BD FACScalibur (BD Biosciences).

### Cell cycle assay

For analysis of cell cycle phase distribution, 2×10^6^ cells were plated in dishes and then treated with indicated concentrations of ribavirin (0∼100 µg/ml) for 24 h, or with the 100 µg/ml ribavirin at indicated time points (0–48 h). Afterwards, the cells were trypsinized, collected, and fixed with 70% ethanol for 30 min. The fixed cells were washed by cold PBS, resuspended in 1 ml of PBS containing 1 mg/ml RNase and 50 µg/ml propidium iodide, and then incubated in the dark for 30 min at room temperature. Finally, the cells were analyzed by BD FACSCalibur. The percentage of cells in G1, S, and G2/M phase of cell cycle and the percentage of cells in sub-G1 (apoptosis) peak were calculated by eliminating the debris effect using CellQuest software (BD Biosciences, San Jose, CA).

### p53 half-life

HCV replicon cells JFH1/HepG2 were grown in DMEM devoid of L-methionine for 3 h before exposure to 200 µCi/ml of [S^35^]-methionine. For assessment of ribavirin effect on p53 half-life, the cells were first labeled with [S^35^]-methionine for 4 h. The culture medium was replaced with a fresh medium, and then these cells were treated with ribavirin for 1 to 16 h. Cellular p53 was immunoprecipitated at the indicated time.

### Immunoblot and antibodies

Immunoblot analysis was performed according to standard procedures. Polyclonal antibodies against phospho-p53 at Ser15 (p53ser15∼*pi*), phospho-ERK1/2 (Thr202/Tyr204) were obtained from Cell signaling Technology, Inc. Monoclonal antibodies against p53, p21, cyclin E, cdk2, Mdm2 were purchased from Santa Cruz Biotechnology, and the HCV NS3 antibody from LTK Biolaboratories (Taiwan). The intensity of the bands was quantitated by densitometry.

### Plasmid construction and transfection

The plasmid containing three copies of the p53 consensus binding sites (p53BS) and the p21- Luc reporter were gifts from Professor Lin [44]. The p53BS-Luc reporter was constructed by inserting the three copies of p53BS into the pGL3-Luc vector. For a transient reporter assay, 8×10^5^ HepG2 and Hep3B cells were seeded in a plate one day before the transfection. Twenty-four hours later, cells were transfected with a total of 4 µg DNA, including 3 µg of the p53BS-Luc reporter, p21-Luc reporter, pcDNA3.1WT-p53, pcDNA3.1mt-(Y220C)-p53 or the reference vector pGL3-Luc plasmid combined with 1 µg the internal control pRL-TK vector by the Arrest-In transfection reagent. Luciferase assays were performed 24 h after transfection and ribavirin treatment using the Dual Luciferase Reporter Kit. Fold induction was calculated by dividing the firefly luciferase activity, normalized to the Renilla luciferase activity, by that observed in the reference vector pGL3-Luc sample without ribavirin treatment.

### ERK1/2 siRNA and transfection

SignalSilence pool ERK1/2 MAP kinase siRNA and scrambled control siRNA were purchased from Cell Signaling Technology. Cells were transfected with ERK1/2-siRNA or scrambled-siRNA using the Arrest-In transfection reagent.

### Lentiviral vector-mediated shRNA knockdown of p53

The p53-shRNA and scrambled-shRNA were obtained from RNAi Core Facility of Academia Sinica in Taiwan. Lentiviral vectors with p53 or scrambled shRNA were prepared in 293FT cells following the established protocols [32], and utilized to transduce HCV replicon cells twice.

### Quantitative real-time RT-PCR assays

The SuperScript cDNA system (Invitrogen) was used to reverse transcribe the RNA template into cDNA for the subsequent PCR amplification. Quantitative PCR was performed with the LightCycler Instrument, and the primers used in this study have been previously described [32]. The results were analyzed with the LightCycler analysis software.

### Statistical analysis

Data were expressed as means ± standard errors of means (s.e.m). The difference between the control and ribavirin-treated cells was evaluated using a Student's *t*-test *and p* value less than 0.05 was considered statistically significant. To determine the effect of p53 in ribavirin-treated cells, we tested the dosage effect of ribavirin in the presence or absence of p53 by two-way ANOVA.

## Supporting Information

Figure S1 **Ribavirin enhances the Mdm2 and p21 RNA levels expression.** Total RNAs were extracted from HepG2 cells with Trizol. The SuperScript cDNA system was used to reversely transcribe the RNA template into cDNA for subsequent PCR amplification. Quantitative PCR was performed using an ABI PRISM 7700 Sequence Detection System. The PCR reaction mixture (50 µl) contained 25 µl of 2× TaqMan Universal PCR Master Mix, 300 nM primers, 200 nM TaqMan probe, 1 µl of cDNA sample and water. The thermal cycling conditions comprised the initial steps at 50°C for 2 min and at 95°C for 10 min, followed by 40 cycles at 95°C for 15 s and at 60°C for 1 min. In order to compare data under the same conditions, data of the target genes were normalized to an internal housekeeping gene, glyceraldehyde-3-phosphate dehydrogenase (GAPDH), for which data was obtained using TaqMan GAPDH control reagents. The data represent means ± s.e.m of triplicate experiments.
(TIFF)

Figure S2 **Reduction of the ribavirin-induced p53 activation and HCV replication by inhibition of ERK1/2 phosphorylation using PD98059.** Suppression of ERK1/2 phosphorylation inhibits p53 functions in (**A**) HepG2 cells and (**B**) HCV replicon cells (JFH1/HepG2), and cells were pretreated with or without 100 µM PD98059 for 4 h, followed by treatment with or without 30 µg/ml ribavirin for 24 h. Immunoblot analysis for phosphorylated ERK1/2 (ERK1/2∼*pi*), phosphorylated p53 at Ser15 (p53ser15∼*pi*), p53, Mdm2 and HCV NS3 were performed. Each analysis was representatives of 4 independent experiments. Control: ribavirin untreated, RBV: ribavirin, PD: PD98059, RBV/PD: Ribavirin plus PD98059.
(TIFF)

Figure S3 **The effects of wild-type p53 (Flag-p53-WT) and mutant p53 (Flag-p53-Y220C) on HCV replication in JFH1/HepG2 cells.** Replication of HCV was determined by (**A**) Western blotting and (**B**) quantitative RT-PCR. Each result represents the mean ± s.e.m of 4 independent measurements and is considered statistically significant at *P*<0.05. (**C**) Viability of the transfected cells was checked by the MTT assay, which was independently repeated 4 times.
(TIFF)

Table S1 **The influence of p53 in the antiviral activity on the ribavirin treated cells.** We compared the dose-dependent suppressive activity of ribavirin in both scrambled-shRNA and p53-shRNA treated cells using a two-way ANOVA test.
(TIFF)
