# Supplementary figures and images for: Ribavirin Enhances the Action of Interferon-α against Hepatitis C Virus by Promoting the p53 Activity through the ERK1/2 Pathway
Source: PLoS One. 2012 Sep 4;7(9):e43824. doi: 10.1371/journal.pone.0043824 (PMC3433463; doi:10.1371/journal.pone.0043824)

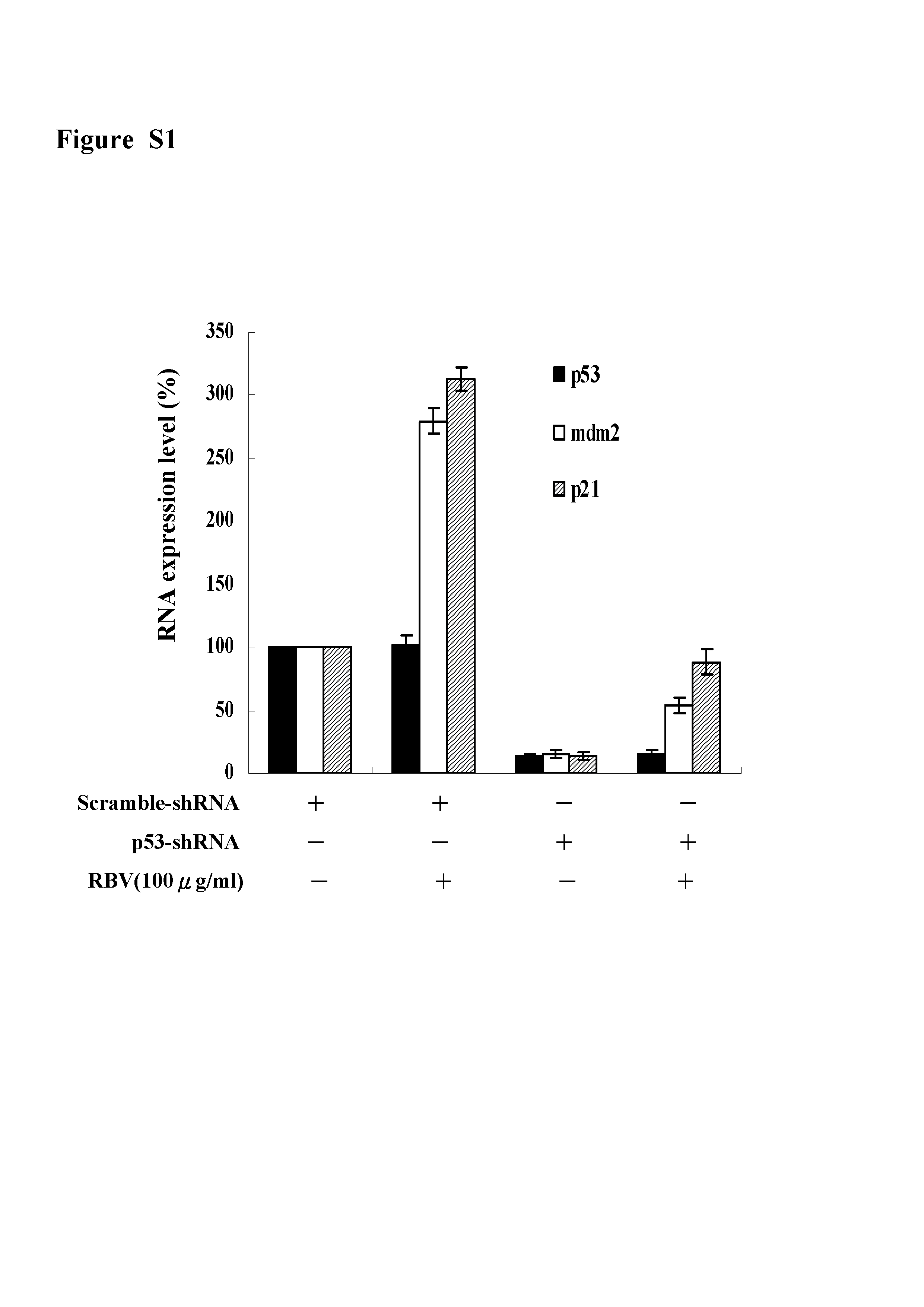

Supplement: Figure S1 — Ribavirin enhances the Mdm2 and p21 RNA levels expression. Total RNAs were extracted from HepG2 cells with Trizol. The SuperScript cDNA system was used to reversely transcribe the RNA template into cDNA for subsequent PCR amplification. Quantitative PCR was performed using an ABI PRISM 7700 Sequence Detection System. The PCR reaction mixture (50 µl) contained 25 µl of 2× TaqMan Universal PCR Master Mix, 300 nM primers, 200 nM TaqMan probe, 1 µl of cDNA sample and water. The thermal cycling conditions comprised the initial steps at 50°C for 2 min and at 95°C for 10 min, followed by 40 cycles at 95°C for 15 s and at 60°C for 1 min. In order to compare data under the same conditions, data of the target genes were normalized to an internal housekeeping gene, glyceraldehyde-3-phosphate dehydrogenase (GAPDH), for which data was obtained using TaqMan GAPDH control reagents. The data represent means ± s.e.m of triplicate experiments. (TIFF) [file pone.0043824.s001.tiff]

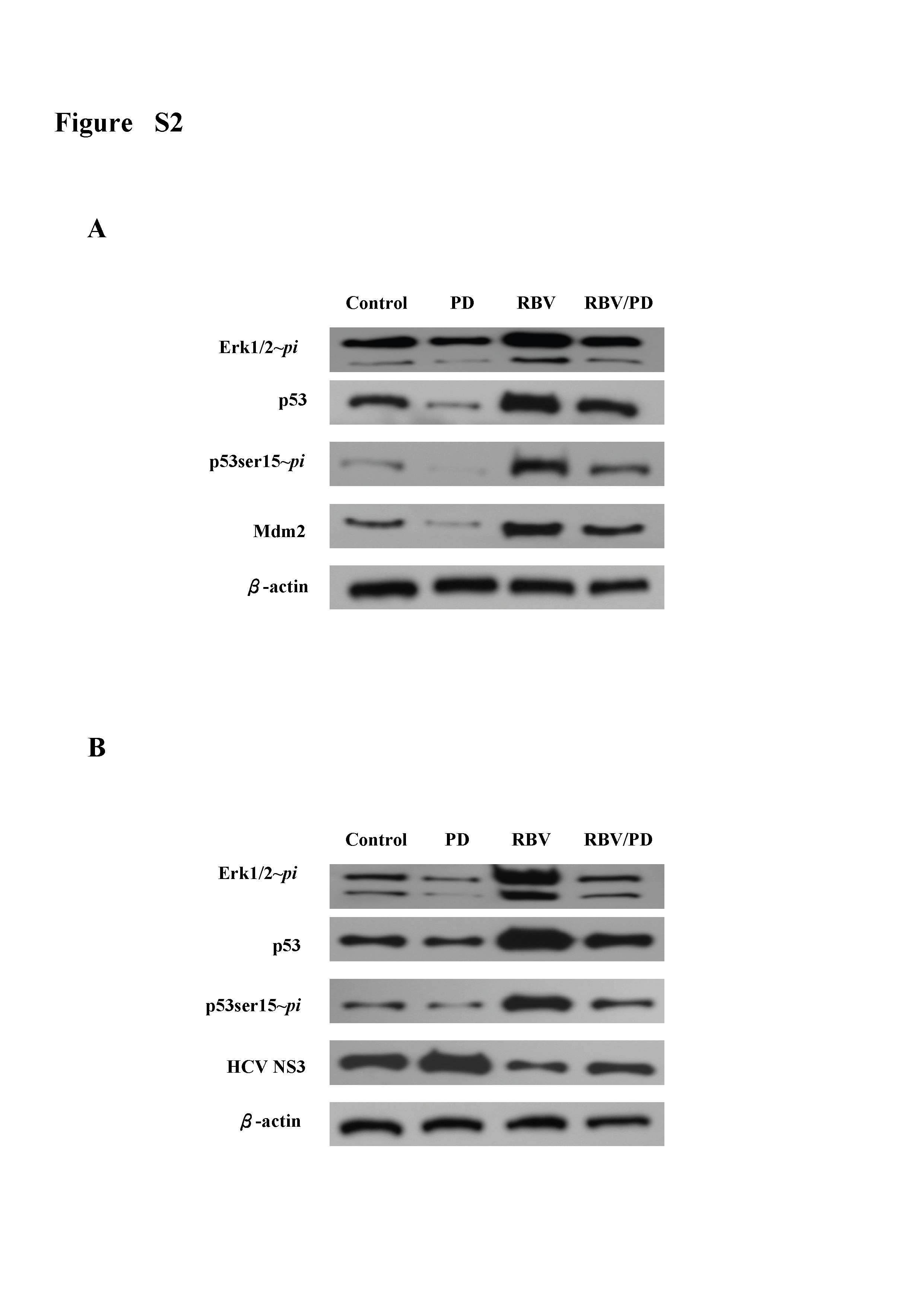

Supplement: Figure S2 — Reduction of the ribavirin-induced p53 activation and HCV replication by inhibition of ERK1/2 phosphorylation using PD98059. Suppression of ERK1/2 phosphorylation inhibits p53 functions in (A) HepG2 cells and (B) HCV replicon cells (JFH1/HepG2), and cells were pretreated with or without 100 µM PD98059 for 4 h, followed by treatment with or without 30 µg/ml ribavirin for 24 h. Immunoblot analysis for phosphorylated ERK1/2 (ERK1/2∼pi), phosphorylated p53 at Ser15 (p53ser15∼pi), p53, Mdm2 and HCV NS3 were performed. Each analysis was representatives of 4 independent experiments. Control: ribavirin untreated, RBV: ribavirin, PD: PD98059, RBV/PD: Ribavirin plus PD98059. (TIFF) [file pone.0043824.s002.tiff]

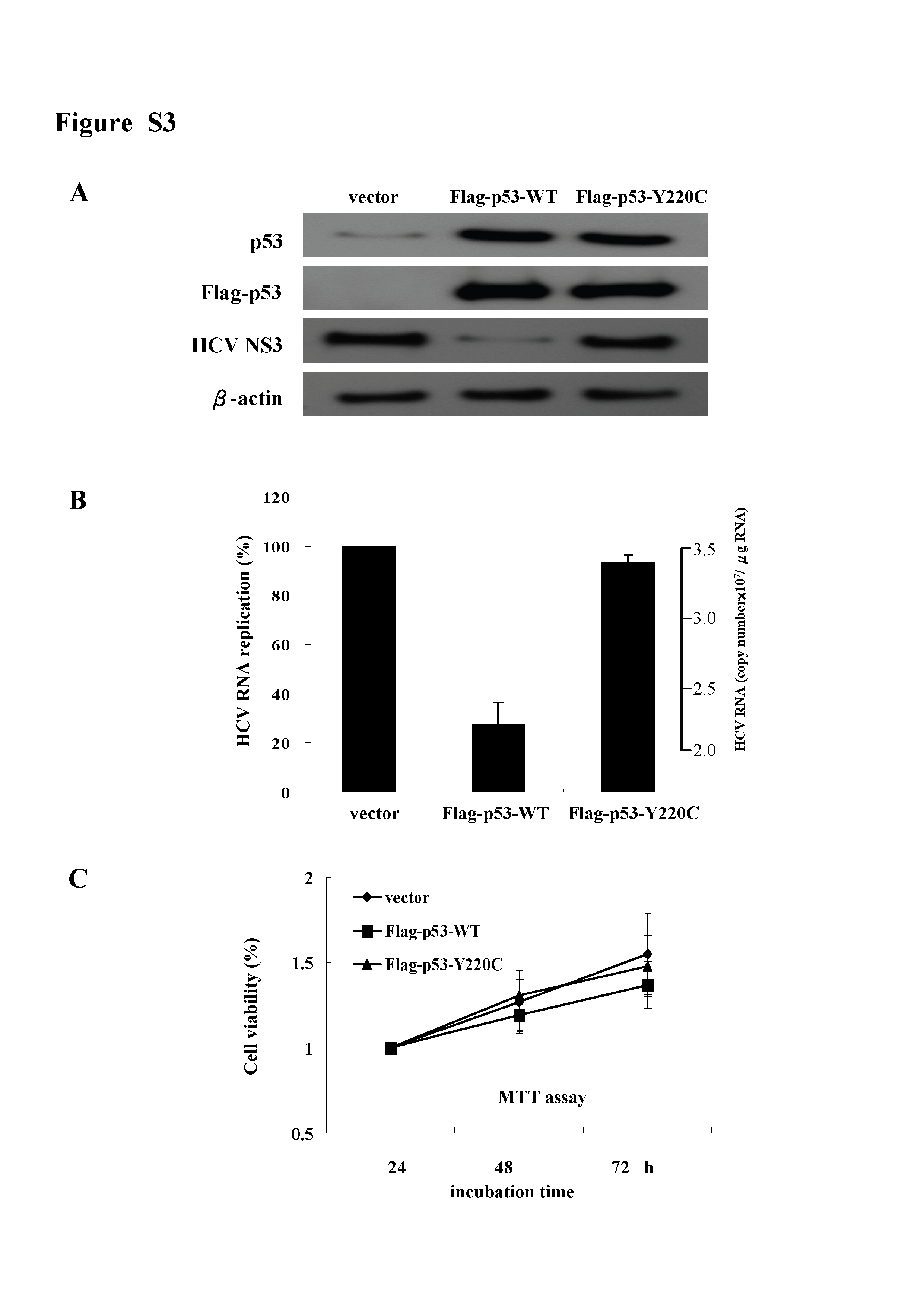

Supplement: Figure S3 — The effects of wild-type p53 (Flag-p53-WT) and mutant p53 (Flag-p53-Y220C) on HCV replication in JFH1/HepG2 cells. Replication of HCV was determined by (A) Western blotting and (B) quantitative RT-PCR. Each result represents the mean ± s.e.m of 4 independent measurements and is considered statistically significant at P<0.05. (C) Viability of the transfected cells was checked by the MTT assay, which was independently repeated 4 times. (TIFF) [file pone.0043824.s003.tiff]

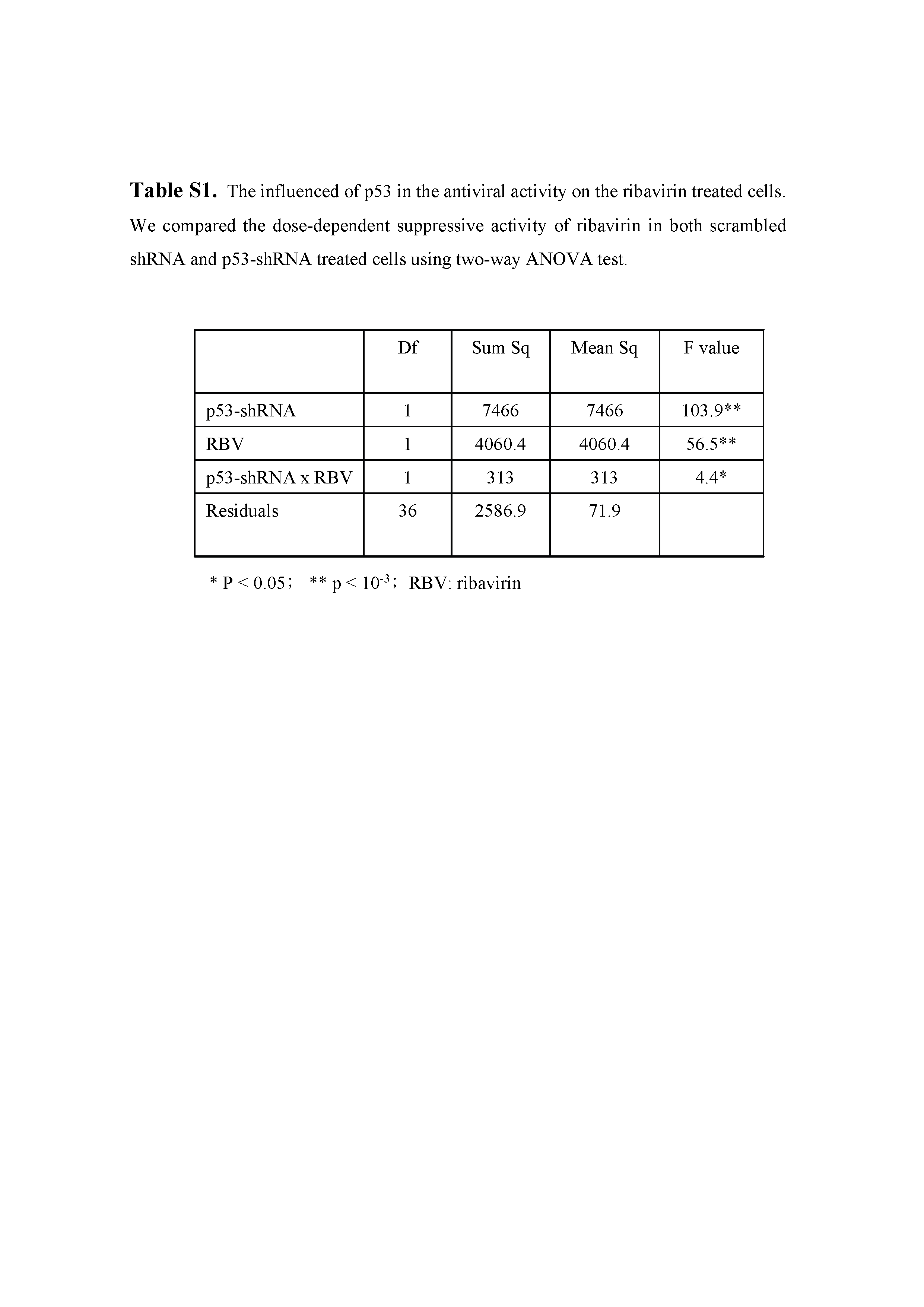

Supplement: Table S1 — The influence of p53 in the antiviral activity on the ribavirin treated cells. We compared the dose-dependent suppressive activity of ribavirin in both scrambled-shRNA and p53-shRNA treated cells using a two-way ANOVA test. (TIFF) [file pone.0043824.s004.tiff]
